# Highly efficient soluble expression, purification and characterization of recombinant Aβ42 from *Escherichia coli*†

**DOI:** 10.1039/c8ra00042e

**Published:** 2018-05-21

**Authors:** Longgang Jia, Wenjuan Wang, Jinzhao Shang, Wenping Zhao, Wei Wei, Ying Wang, Li Li, Fuping Lu, Fufeng Liu

**Affiliations:** State Key Laboratory of Food Nutrition and Safety (Tianjin University of Science & Technology) Tianjin 300457 P. R. China fufengliu@tust.edu.cn +86-22-60602298 +86-22-60602717; Key Laboratory of Industrial Fermentation Microbiology (Tianjin University of Science & Technology), Ministry of Education Tianjin 300457 P. R. China lfp@tust.edu.cn +86-22-60602298 +86-22-60602271; Tianjin Key Laboratory of Industrial Microbiology, Tianjin University of Science & Technology Tianjin 300457 P. R. China; College of Biotechnology, Tianjin University of Science & Technology Tianjin 300457 China; College of Marine and Environmental Sciences, Tianjin University of Science & Technology Tianjin 300457 P. R. China

## Abstract

Aggregation of amyloid-β protein (Aβ) is hypothesized to be a seminal neuropathological event in Alzheimer's disease (AD). Recombinant expression and purification of Aβ represents a common basis for investigating the molecular mechanisms of amyloid formation and toxicity. Herein, we report a novel high-yield expression and purification method for Aβ42 based on fusion with maltose binding protein (MBP) followed by the soluble polypeptide linker (NANP)_3_ and a modified tobacco etch virus (TEV) cleavage site before the Aβ42. We obtained a final yield of ∼18 mg L^−1^ of recombinant Aβ42 that was confirmed by SDS-PAGE, protein immunoblotting and MALDI-TOF. Finally, thioflavin T fluorescence and atomic force microscopy revealed that the recombinant Aβ42 aggregated into long, branched fibrils. Furthermore, the aggregates of the recombinant peptide had a strong cytotoxic effect on PC12 cells. The method described here can therefore be used to efficiently express the soluble fusion protein MBP-Aβ42 and obtain high-purity Aβ42 peptide, which can be used to understand the molecular mechanism of Aβ42 fibrillization and screen new candidate drugs for AD.

## Abstract 2

A novel high-yield expression and purification method for Aβ42 based on a fusion with maltose binding protein followed by the soluble polypeptide linker (NANP)_3_ and a modified tobacco etch virus cleavage site before the Aβ42 was developed.

## Graphical abstract

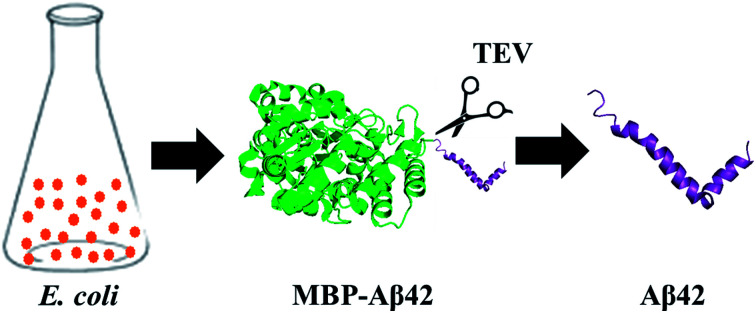

## Introduction

Aggregation of amyloid proteins into insoluble fibrils can cause many neurodegenerative diseases, including Alzheimer's (AD) and Parkinson's disease, but also type-II diabetes.^1^ AD is one of the neurodegenerative diseases that presently severely affect tens of millions of elderly patients around the world, causing a great economic burden to families and society.^2^ According to the data published in 2016 by the Alzheimer's Association International Conference, about 46.8 million people in the world were living with AD in 2015, and this number is doubling every 20 years. Moreover, the incidence rate of AD is even increasing, so that it is estimated that in 2050 there will be more than 130 million AD patients worldwide.^3^ It was estimated that the cost of medical care for the global population of AD patients in 2015 was about $818 billion, about 1.1% of global GDP.^4^ Therefore, exploring the etiology of AD and finding anti-AD drugs has become the focus of researchers all over the world.^5^

One of the pathological features of AD is the accumulation of extracellular senile plaques in the patients' brains.^6^ The major components of neuritis plaques in AD patients' brains are various forms of aggregates of amyloid-β protein (Aβ). Aβ peptides containing 39–43 amino acids are produced by the sequential hydrolysis of amyloid precursor protein by both β- and γ-secretases.^6^ Among them, Aβ40 and Aβ42 are the two most common components.^7^ Since the C-terminus of Aβ42 contains two vicinal hydrophobic amino acids, Ile-Ala, Aβ42 aggregates rapidly, and the resulting aggregates are the most cytotoxic species.^8–10^ Therefore, Aβ42 has become a focus of research.

Currently, Aβ42 used in research is mainly produced by solid-phase chemical synthesis.^11,12^ Although this method is efficient, rapid and inexpensive, the variable amounts of intrinsic impurities including residual amino acids, fragments and reagents from the solid-phase synthesis process seriously affect the aggregation properties and the corresponding toxicity of Aβ42.^13,14^ For example, Adams *et al.* identified the Aβ42Δ39 species as the major peptide contaminant responsible for limiting both cytotoxicity and fibrillation of chemically synthesized Aβ42.^15^ In addition, it is difficult to purify Aβ42 from neuronal cells in large amounts due to its high intrinsic hydrophobicity, proneness to aggregation, and extremely low abundance.^16^ Consequently, heterologous expression has become the main method to produce Aβ42. Some studies have used genetic engineering methods and subsequent expression and purification steps to obtain various types of recombinant amyloid-β peptides including Aβ(M1-40) and Aβ(M1-42),^17^ as well as ^15^N- and ^13^C-isotope labeled Aβ40 (ref. 18) and ^15^N isotope-labeled Aβ42 (ref. 19 and 20) in *E. coli*. Moreover, preparative SDS-PAGE was also used to purify the recombinant Aβ42 and pGlu-Aβ42.^21^ In addition to *E. coli*, *P. pastoris* was also used to express Aβ42.^22^ However, the disadvantages of low yield, complex purification steps and insufficient purity of Aβ are still present. Moreover, there are some intrinsic shortcomings, such as the addition of a methionine to the peptide and difficult purification. Moreover, prolonged fermentation protocols may result in the accumulation of large amounts of Aβ42 fibrils, which is not conducive to the later purification and aggregation analysis.

Maltose-binding protein (MBP) is a commonly used fusion tag that can improve the yield and soluble expression of target proteins, and has been used in the recombinant expression of several hydrophobic proteins.^23,24^ Because of the strong affinity of MBP for amylose, the MBP-fused protein can be specifically purified over amylose resin to 70–90% purity.^25^ In order to obtain pure Aβ42, a protease cutting site is needed between the fusion tag and Aβ42. Proteases in common use include tobacco etch virus protease (TEV),^20^ thrombin,^26^ and enterokinase^27^*et al.* The TEV protease is widely used in the separation of fusion proteins, in specific protein markers for genomics or proteomics, and segregation analysis.^20,28^ However, after cleavage with TEV, the recombinant Aβ peptide would normally carry an additional residue from the cutting site, which may have an influence on its aggregation properties.^28^

In this work, a generally applicable, efficient protocol for the production of recombinant Aβ42 without any extra residues with MBP as a fusion protein in *E. coli*, and an efficient and simple procedure to obtain pure Aβ42 was developed. The clone expresses an Aβ42 fusion protein containing an MBP tag, the rigid and soluble (NANP)_3_ linker, a modified recognition site for TEV protease which changes the ENLYFQG sequence to ENLYFQ, and full-length Aβ42. After identification *via* western blot analysis and MALDI-TOF, the target protein was proved to be Aβ42. We further characterized the Aβ42 by thioflavin T (ThT) binding assays, AFM and cytotoxicity experiments, demonstrating its suitability for widespread use in biological research.

## Materials and methods

### Materials

The expression vector pMAL-c2x was purchased from Novagen Inc. (Darmstadt, Germany). *E. coli* JM109 was used as the host for plasmid construction and molecular cloning of the candidate gene. *E. coli* BL21 (DE3) was used as the host for heterologous expression of the candidate gene. Both of them were obtained from Invitrogen Inc. (Carlsbad, USA) and grown at 37 °C in Luria-Bertani (LB) medium (10 g L^−1^ tryptone; 5 g L^−1^ yeast extract; 10 g L^−1^ NaCl and 15 g L^−1^ agar; pH 7.0). Restriction enzymes and T4 DNA ligase were purchased from Takara Inc. (Dalian, China) and used with the provided buffer according to supplier's recommendations. QIAprep Spin MiniPrep Kit and amylose resin were purchased from Qiagen Inc. (Hilden, Germany). Aβ42 gene was codon-optimized and synthesized by GENEWIZ Inc. (Suzhou, China). Primers and sequence analyses of the constructions were performed by BGI Inc. (Shenzhen, China). Commercial Aβ42 (>95%) was purchased from GL Biochem Ltd. (Shanghai, China) Dulbecco's modified Eagle's medium and fetal bovine serum were purchased from Gibco Invitrogen Inc. (Grand Island, NY, USA). The PC12 cell line was obtained from National Infrastructure of Cell Line Resources of China. Unless noted, all other reagents and chemicals were of the highest purity available from local sources.

### Construction of pMAL-Aβ42 expression vector

The codon-optimized DNA amino acid sequences of Aβ42 were performed to prefer for *E. coli*. The Aβ42 DNA fragment was synthesized in pUC57 vector as pUC57-(NANP)_3_-TEV-Aβ42. The target fragment was digested from the plasmid with EcoRI and HindIII and extract with Gel Extraction Kit. Then the fragment was ligated into pMALc2x vector with the same cohesive end for 4 h at 16 °C. The ligated product was transformed into *E. coli* JM109 competent cells. After culturing in 37 °C for 12 h, positive transformants were picked for colony PCR validation, and the plasmid was extracted for identification by gene sequencing. The identified plasmid was named pMAL-Aβ42.

### Expression, purification of MBP-Aβ42 fusion protein and digestion by TEV protease

The recombinant plasmid pMAL-Aβ42 was transformed into *E. coli* BL21 (DE3). Single colony was picked and cultured in 5 mL LB medium at 37 °C overnight, and inoculated to 200 mL fresh LB medium with 1% inoculation until the OD600 to 0.6. After adding a final concentration of 0.5 mM isopropyl-β-d-thiogalactoside, the recombinant BL21-Aβ42 culture was incubated at 16 °C for 16–18 h. The cell pellets were harvested (8000 rpm, 10 min, 4 °C) and suspended in 20 mL of column buffer (20 mM Tris–HCl, pH 7.4, 200 mM NaCl, 1 mM EDTA, 1 mM DTT). A final concentration of 30 μg mL^−1^ lysozyme and 1% (v/v) phenylmethanesulfonyl fluoride were added to the solution. After incubating in ice bath for 30 min and submitting to sonication (15 cycles of 10 s), the mixture was centrifuged at 12 000 rpm at 4 °C for 40 min. The supernatant and precipitate were separated and analyzed by SDS-PAGE. The fusion protein in the supernatant was purified by affinity chromatography with amylose resin and quantitated by Nanodrop 2000 (Thermo Fisher Scientific, Wilmington, USA). 1 mg of the above purified fusion protein was mixed with 4 μL of 250× TEV buffer, 2 μL or 4 μL TEV enzyme (5 U μL^−1^) and make up to 1 mL volume with buffer (50 mM NaH_2_PO_4_, 150 mM NaCl). Followed by incubating at 23 °C for 3 h, 6 h or 12 h, the products of TEV digesting were analyzed by 12% SDS-PAGE.

### SDS-PAGE analysis

20 μL of protein sample was mixed with 5 μL Laemmli Sample buffer (Bio-Rad) containing 10% β-mercaptoethanol, and denatured at 100 °C for 10 min. Then the protein was separated by 12% polyacrylamide gels. Electrophoresis was run for 30 min at 60 V and then 120 V for nearly 1 h in tris-glycine SDS buffer. Gels were stained with Coomassie brilliant blue R250 and de-stained with water.

### Purification of recombinant Aβ42 using size-exclusion chromatography

According to the optimal conditions for digestion, aliquot amount of the fusion protein was taken for digesting. The TEV cleavage product was concentrated to 500 μL and filtered through a 0.22 μm syringe filter. A GE gel filtration column, Superdex 200, was used to separate Aβ42 with a column volume of 30 mL and the mobile phase was 20 mM Tris–HCl, pH 7.4, 150 mM NaCl, 1 mM DTT. The effluent phase was collected according to the peak time. The purified Aβ42 was detected by Tricine-SDS-PAGE gel kit (Solarbio, Beijing, China). At last, the product was lyophilized and stored at −80 °C.

### Western blot and MALDI TOF mass spectrometry

The recombinant Aβ42 was separated by Tricine-SDS-PAGE, and the gel was transferred onto a polyvinylidene fluoride membrane at 100 V for 30–60 min using a Bio-Rad mini Trans-Blot electrophoretic transfer for western blot analysis.^29^ The obtained membrane was sealed in 5% degreased milk powder solution and incubated for 1 h. Then the blotted membrane was rinsed with TBST buffer (20 mM Tris–HCl, pH 8.0, 150 mM NaCl, 0.05% (v/v) Tween 20) for four times. After probing with the rabbit anti-Aβ42 antibody 6E10 (1 : 1000 dilution, Abcam) in 4 °C overnight, the membrane was washed with TBST buffer for four times and then incubated in the goat anti-rabbit IgG secondary antibody (1 : 1000 dilution, Beijing Zhongshan Golden Bridge Biotechnology Co. Ltd., China) at room temperature for another 2–3 h. Following four times washes with TBST buffer, the membrane was visualized with an infrared laser imaging system (LI-COR, USA). The target band of Aβ42 from Tricine-SDS-PAGE gel was cut and sent to the Tianjin International Biomedical Research Institute for MALDI TOF mass analysis.

### Thioflavin T fluorescence assay

Aβ42 was dissolved in 20 mM NaOH and centrifuged at 16 000 g for 20 min to remove the preformed aggregates. Then, the supernatant of Aβ solution was immediately diluted to a final 25 μM use 10 mM PBS buffer (pH 7.4, 100 mM NaCl). Together with 25 μM ThT, the freshly prepared Aβ monomers were incubated at 37 °C for 72 h. The ThT fluorescent assay was performed by a fluorescence plate reader (Infinite 200PRO Laboratories, TECAN, Austria). The wavelengths of the excitation and emission were 440 nm and 485 nm, respectively. Three measurements were performed and all data represent the mean ± standard deviation.

### Atomic force microscopy

At given time points (0 and 3 d in this study), 20 μL of Aβ solution was deposited onto a freshly cleaved mica sheet. After incubation for 5 min at room temperature, the mica sheet was rinsed three times with deionized water to remove salts and loosely bound Aβ species. Excess water was removed with a gentle stream of nitrogen. Previous studies have shown that the morphology of Aβ42 fibrils in the liquid^30^ is similar to the one observed in the air.^31^ Moreover, most studies performed AFM experiments in air to observe the morphology of fibrils formed by Aβ.^32,33^ Therefore, tapping mode AFM imaging was performed in air using a Multimode 8 AFM (Bruker, USA) with a Scanasyst-Air silicon probe tip (115 μm cantilever, spring constant: 0.4 N m^−1^, tip radius: 2 nm, resonance frequency: ∼70 kHz). Scanning parameters were as follows: peak force setpoint, 0.3–1.0 V; scan rate, 0.8–2.0 Hz. During the AFM imaging, we set the scan size to 2 μm × 2 μm, and the resolution was set to 1024 lines. Further nano-mechanical measurements were conducted in PF-QNM mode and the tip was initially calibrated on calibration sample (sapphire surface) to obtain the deflection sensitivity. The cantilever spring constant and tip radius were further calibrated following the operation manual. All AFM images were analyzed using Nanoscope analysis 1.8 software (Bruker, USA).

### Cytotoxicity assay

*In vitro* cytotoxicity assay was performed using PC12 cell line. PC12 cells were cultured in Dulbecco's modified Eagle's media supplemented with 10% fetal bovine serum. The cells were plated at a density of ∼5000 cells per well in 96-well plates with 90 μL of fresh medium. After incubated for 24 h, the aged Aβ42 aggregates (10 μL each well) were added into the plates, and the cells were incubated for another 48 h. Then, 10 μL of MTT store solution (5.0 mg mL^−1^) was added into each well, and the plates were incubated for another 4 h. After removing the culture medium, precipitated cells were lysed using DMSO, and the absorbance at 570 nm was measured using a Microplate Reader (Infinite 200PRO Laboratories, TECAN, Austria). The wells containing medium only were subtracted as the background from each reading. The cell viability data were normalized as a percentage of the control group.

## Results and discussion

### Cloning and plasmid construction for MBP-Aβ42 fusion protein expression

**Fig. 1:**
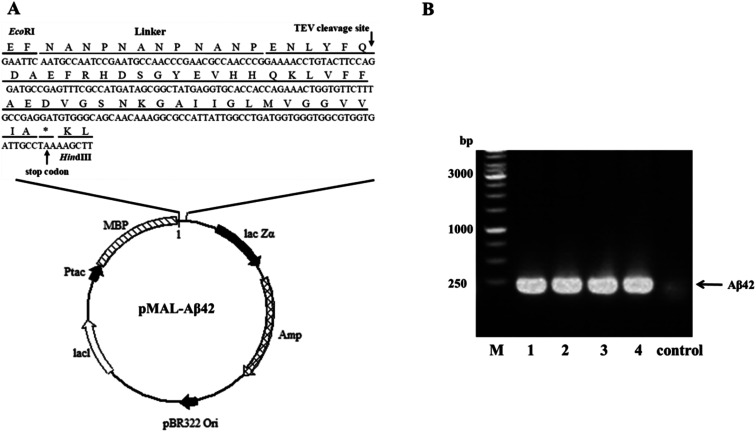
Construction of the MBP-Aβ42 fusion protein expression vector. (A) General diagram of the expression vector pMAL-Aβ42. (B) Identification of the transformants *via* colony PCR. M: 1 kb marker; 1–4: pMAL-Aβ42 transformants; control: JM109 wild type.

A schematic of the plasmid pMAL-Aβ42 that was used for the expression of the MBP-Aβ42 fusion protein is shown in Fig. 1A. In order to obtain the authentic Aβ42 peptide, the Aβ42 gene was inserted downstream of the MBP-encoding DNA in the same open reading frame, by ligating it between the EcoRI and HindIII restriction sites in the multiple cloning site of the plasmid pMALc2x. Between MBP and Aβ42, there was a rigid and soluble linker (NANP)_3_ and a modified recognition sequence for TEV (from ENLYFQ↓G to ENLYFQ↓; the arrow indicates the cleavage site) which can release the authentic Aβ42 without leaving any excess residues. Firstly, the codon usage of the (NANP)_3_-TEV-Aβ42 fragment was optimized for *E. coli*, and the DNA sequence (ESI Table S1†) was obtained by synthesis, and was delivered cloned into the plasmid pUC57-(NANP)3-TEV-Aβ42. The fragment encoding (NANP)_3_-TEV-Aβ42 was double-digested using EcoRI/HindIII and sub-cloned into the pMALc2x expression vector. The correct assembly of the recombinant expression vector was confirmed by PCR using the primer pair EcoRI-Aβ42-F/HindIII-Aβ42-R (ESI Table 1†). As shown in Fig. 1B, PCR product of the expected size (approximately 200 bp) was generated from four positive transformants, while the JM109 wild-type control showed no clear band at the corresponding location. Furthermore, the orientation and sequence of the gene fragment was verified by DNA sequencing (data not shown). These results proved that the designed expression vector pMAL-Aβ42 was successfully constructed as intended.

### Expression and purification of the MBP-Aβ42 fusion protein

**Fig. 2:**
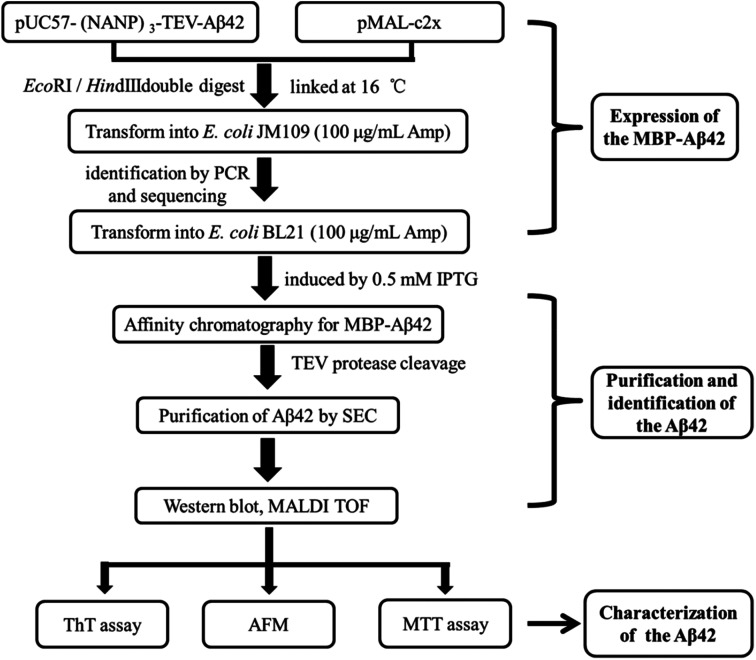
Flow chart of the construction, expression, purification, identification and characterization protocol for the recombinant Aβ42.

**Fig. 3:**
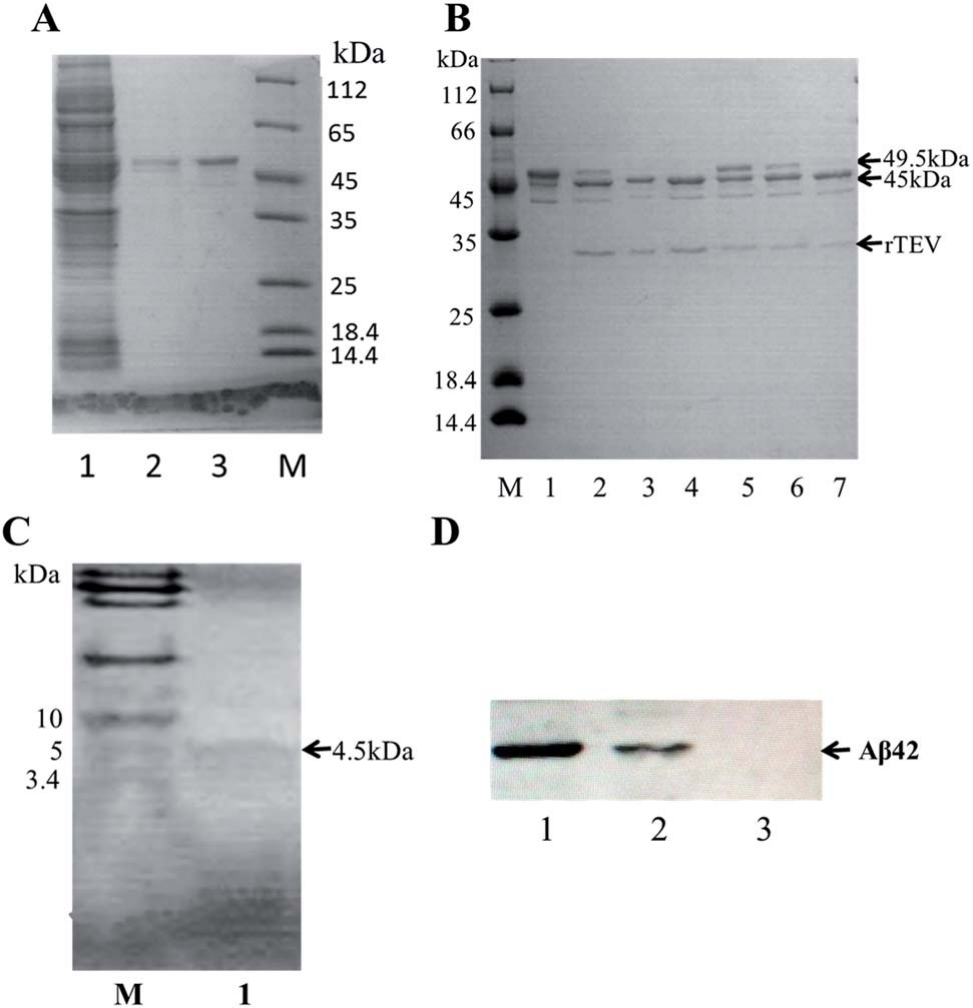
Purification, enzymatic digestion analysis and identification of recombinant Aβ42. (A) SDS-PAGE analysis of the MBP-Aβ42 purification process. Lane 1: flow-through; lane 2: wash buffer (column buffer); lane 3: elution buffer (column buffer with 10 mM maltose); M: protein marker. (B) Optimization of the TEV digestion reaction. M: protein marker; lane 1: MBP-Aβ42 fusion protein; lanes 2–4: incubation for 3, 6, and 12 h with 4 μL of a 5 U μL^−1^ TEV solution; lanes 5–7: incubation for 3, 6, and 12 h with 2 μL of the same TEV solution. (C) Tricine-SDS-PAGE of recombinant Aβ42 purified *via* size-exclusion chromatography; (D) western blot analysis of recombinant and chemically synthesized Aβ42. Protein samples were separated on 12% SDS-PAGE, transferred to a polyvinylidene fluoride membrane and probed with the monoclonal anti-Aβ42 antibody 6E10. Lanes 1–3: recombinant Aβ42, chemically synthesized Aβ42, total protein of *E. coli* BL21 containing pMAL-c2x vector and induced for 2 h.

A schematic of the expression and purification process of the Aβ42 peptide is shown in Fig. 2. The plasmid pMAL-Aβ42 was first transferred into the expression strain BL21. After cultivation on a plate at 37 °C overnight, a single colony of the recombinant bacteria was used to inoculate LB medium, and induced with isopropyl-β-d-thiogalactoside. The cells were collected and submitted to sonication. The supernatant and sediment were analyzed by SDS-PAGE, which showed that the MBP-Aβ42 fusion protein was present in the supernatant, demonstrating its successful soluble expression (ESI Fig. 1†). The supernatant of the cell lysate was separated using affinity chromatography with amylose resin. After washing twice with column buffer, the MBP-Aβ42 fusion protein was eluted with column buffer containing 10 mM maltose. The flow-through, wash, and elution fractions were analyzed by SDS-PAGE as shown in Fig. 3A. It was clear that the amounts of the MBP-Aβ42 fusion protein in the flow-through and wash fractions were very low. By contrast, the elution fraction contained mainly MBP-Aβ42 fusion protein, with minor impurities comprising low-molecular-weight proteins. As can be seen in Fig. 3A, the molecular weight of the fusion protein was approximately 49.5 kDa, which was consistent with its predicted molecular weight. The fusion protein yield was estimated to be up to ∼240 mg per liter of culture.

### TEV protease cleavage of the MBP-Aβ42 fusion protein and purification of recombinant Aβ42.

In order to obtain authentic Aβ42 without any additional amino acid residues, the cleavage site of the TEV protease (ENLYFQ↓G) was changed to the 6-amino-acid sequence (ENLYFQ↓). It is known that 90% cleavage efficiency of TEV is retained when the glycine is substituted by aspartate,^34^ which is serendipitously also the first amino acid of Aβ42. Therefore, after the MBP-Aβ42 fusion protein is digested with TEV protease, native-like Aβ42 without any additional residues can be obtained. In order to achieve the best digestion effect, the digestion conditions were optimized by modulating the addition of TEV protease and the digestion time. The cleavage efficiency was estimated by SDS-PAGE and the results are shown in Fig. 3B. The molecular weight of the MBP-Aβ42 was about 49.5 kDa, which can be cleaved into the two fragments MBP and Aβ42 with 45 and 4.5 kDa, respectively. After cleavage for 3 h, two lanes were observed around 49.5 kDa and 45 kDa, corresponding to the MBP-Aβ42 fusion protein and MBP, respectively (Fig. 3A). Since low-molecular-weight proteins are difficult to visualize by the ordinary SDS-PAGE used in the current study, the Aβ42 band cannot be seen in Fig. 3B. However, the effect of TEV cleavage could nevertheless be analyzed qualitatively and quantitatively due to the gradually decreasing 49.5 kDa band of the MBP-Aβ42 fusion protein and increasing 45 kDa band of the MBP tag. We found that TEV cleavage was significantly enhanced by adding more protease. The results showed that the optimal conditions for enzymatic digestion encompassed a reaction temperature of 23 °C, a ratio of the MBP-Aβ42 fusion protein to TEV protease of 1 mg to 4 μL of an 5 U μL^−1^ TEV solution, and a reaction time of 12 h.

The digested product was further purified by size-exclusion chromatography (SEC) to obtain high-purity Aβ42 (Fig. S2†). As shown in Fig. 3C, the purified product was verified by Tricine-SDS-PAGE. A clear single band around 4.5 kDa was visible, indicating a molecular weight that is consistent with the theoretical molecular weight of Aβ42. We obtained approximately ∼18 mg of purified Aβ42 from 1 L of culture, which is a very high yield compared to the other strategies adapted for the affordable production of Aβ peptide.^16,19,20,35^

### Characterization of the purified recombinant Aβ42

The identity of the purified recombinant Aβ42 peptide was further confirmed by immunoblotting. A chemically synthesized Aβ42 peptide and cell lysate of *E. coli* BL21 containing pMAL-c2x vector were used as positive and negative control, respectively. The samples were separated *via* electrophoresis and transferred onto a PVDF membrane. After incubating overnight at 4 °C with the monoclonal rabbit antibody 6E10, which specifically binds to Aβ42,^36^ the membrane was incubated with a goat anti-rabbit IgG secondary antibody for 2–3 h at room temperature, and the membrane scanned by an infrared laser imager. As shown in Fig. 3D, the recombinant Aβ42 obtained in this study was identical to chemically synthesized Aβ42, with both samples showing a single specific band at the same position, while there was no band in the control group comprising BL21 cell lysate. This result proved that the purified product is authentic Aβ42 peptide.

**Fig. 4:**
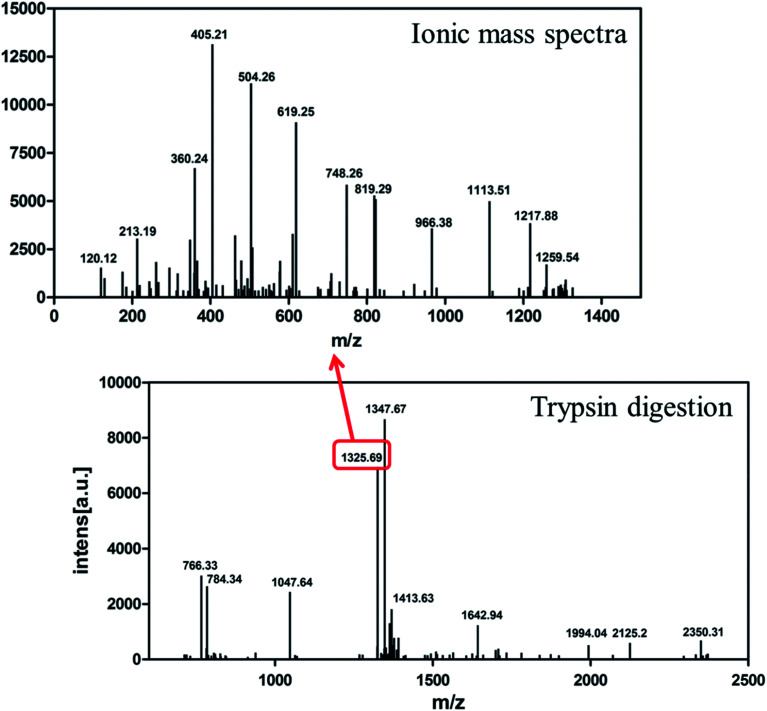
MALDI-TOF/TOF mass spectrometry of recombinant Aβ42. Bottom: mass spectrometry of Aβ42 digested by trypsin. Top: ionic mass spectra of peak 1325.69.

The target band from the gel was cut out and analyzed by MALDI-TOF/TOF mass spectrometry. As shown in Fig. 4, the target product ion spectra derived from the fragmentation of the charged ion at *m*/*z* 1325.69 correspond to the peptide KLVFFAEDVGSNKG (residues 16–29 of Aβ42). Analysis of the secondary ion chromatograms revealed *m*/*z* 504 corresponding to the peptide KLVF (residues 16–19 of Aβ42) and *m*/*z* 966.58 corresponding to the peptide FAEDVGSNKG (residues 19–29 of Aβ42). The mass spectrum therefore corroborated that the target product was indeed authentic Aβ42.

### Biophysical characterization of purified recombinant Aβ42

**Fig. 5:**
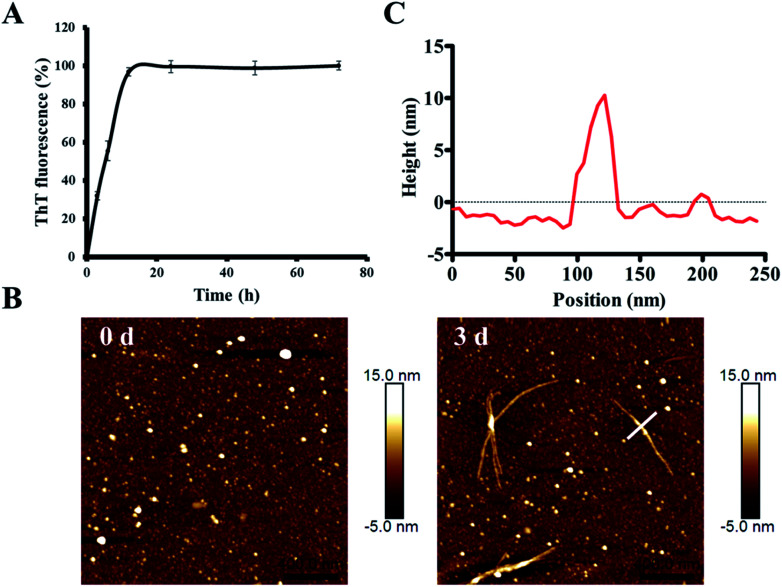
Investigation of the amyloid-forming properties of the recombinant Aβ42 peptide. (A) ThT fluorescence analysis of the aggregation of 25 μM recombinant Aβ42 at 37 °C in 10 mM PBS buffer pH 7.4. (B) Atomic force microscopy images of recombinant Aβ42 species after incubation for 0 and 3 days. (C) Height of the cross sections drawn over the fibril in (B).

In order to characterize the aggregation properties of the purified recombinant Aβ42, we analyzed the amyloid properties by studying its aggregation kinetics, AFM and cytotoxicity. The ThT fluorescence assay is considered a highly sensitive tool for detecting the formation of amyloid aggregates of various amyloidogenic proteins.^37–39^ Therefore, this assay was used to characterize the aggregation kinetics of recombinant Aβ42 as shown in Fig. 5A. When 25 μM recombinant Aβ42 was incubated at 37 °C for 72 h, the ThT fluorescence profile showed an almost negligible lag phase, a fast growth phase within 20 h, and a steady equilibrium phase after 24 h. Fig. 5B shows the AFM images of Aβ42 after 0 and 3 days of incubation, which confirmed that the recombinant Aβ42 formed typical long, branched fibrils with lengths of 200–700 nm. As shown in Fig. 5C, in the height image derived from the cross-section of the mature fiber in Fig. 5B (indicated by the white bar), the height of the fibrils was about 5–15 nm. The corresponding 3D images of the two artifacts are also shown in Fig. S3A,† which present a visual view of the fibrils. Further nano-mechanical properties of the fibrils were analyzed in PF-QNM mode and the adhesion image is shown in Fig. S3B.† The dashed white line marks the position at which the adhesion image was analyzed and the profile of the adhesion force along the fibril is shown in Fig. S3C.† According to the results, the average value of the adhesion force along the fibril was 2.35 ± 0.66 nN.

This observation was consistent with the ThT fluorescence results and our previous studies.^40^ Therefore, both ThT and AFM results indicated that the recombinant Aβ42 showed biochemical behavior consistent with the aggregation characteristics of previously reported recombinant and synthetic Aβ42.^17,40–42^

### Cytotoxicity of recombinant Aβ42 towards PC12 cells

**Fig. 6:**
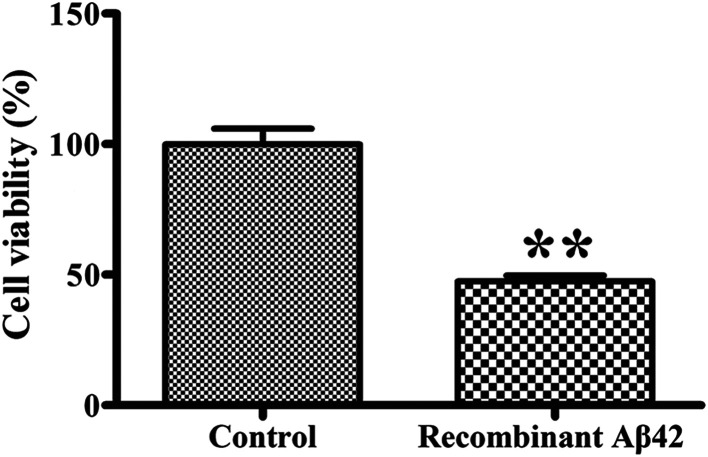
Cytotoxicity of recombinant Aβ42 (3 μM) fibrils toward PC12 cells. After treatment for 48 h, the cells were subjected to the MTT assay. Values are relative to those of control cells mock-treated with complete medium alone. Values represent the means ± SD (*n* = 3). **p < 0.01, compared to the control.

To confirm the cytotoxicity of the recombinant Aβ42, the cell viability of cultures of the PC12 neuronal cell line exposed to it was assessed using the standardized MTT assay. We set the survival of cells mock-treated with PBS buffer only as a basis of 100% to normalize the other data for comparison. As shown in Fig. 6, treatment of the PC12 cells with 3 μM recombinant Aβ42 for 48 h reduced their viability by about 47.5%. These results suggest that the aggregates of the recombinant Aβ42 exhibit strong cytotoxicity, which was expected based on the results of the ThT assay and AFM.

## Conclusions

In this study, we developed a novel method for the efficient expression and purification of recombinant Aβ42 without any additional residues. Herein, an MBP fusion tag was used to improve the solubility and yield of Aβ42. The rigid and soluble linker (NANP)_3_ and the modified TEV protease recognition site ENLYFQ were added between the MBP and Aβ42. After optimization of the TEV cleavage conditions, the MBP tag was removed, yielding 18 mg L^−1^ of authentic Aβ42, which was identified by western blot analysis and MALDI-TOF mass spectrometry. ThT fluorescence and AFM assays demonstrated the great aggregation activity of the recombinant peptide, and the MTT assay demonstrated that the aggregates of the recombinant Aβ42 showed neurotoxicity *in vitro*. Furthermore, the method developed in this study provides us with a biochemical tool to obtain Aβ variants with various lengths or sequences that will certainly facilitate further structural and functional studies in the future. Therefore, the availability of purified Aβ42 could enable new structural, biochemical, and biological insights, as well as cheaper screening of anti-AD drug candidates. Moreover, the recombinant protein could also be used as a reagent for the preparation of monoclonal antibodies against Aβ42, which can also be used in anti-Aβ therapies for AD. Finally, this study also provides a potential strategy for the expression and purification of other amyloid proteins in *E. coli*.

## Conflicts of interest

There are no conflicts of interest to declare.

## Supplementary Material

RA-008-C8RA00042E-s001
