# Supplementary material for: Highly efficient soluble expression, purification and characterization of recombinant Aβ42 from Escherichia coli
Source: RSC Adv. 2018 May 21;8(33):18434–41. doi: 10.1039/c8ra00042e (PMC9087987; doi:10.1039/c8ra00042e)
Supplement: RA-008-C8RA00042E-s001 [file RA-008-C8RA00042E-s001.pdf]

Electronic Supplementary Material (ESI) for RSC Advances.
This journal is © The Royal Society of Chemistry 2018

Supplementary Information

Highly Efficient Soluble Expression, Purification and Characterization of Recombinant Aβ42 from *Escherichia coli*

Longgang Jia,[1,2,3,4] Wenjuan Wang,[1,2,3,4] Jinzhao Shang,[1,2,3,4] Wenping Zhao,[1,2,3,4] Wei Wei,[1,2,3,4] Ying Wang,[1,2,3,4] Li Li,[5] Fuping Lu,[1,2,3,4]* Fufeng Liu[1,2,3,4]*

[1] State Key Laboratory of Food Nutrition and Safety (Tianjin University of Science & Technology), Tianjin, 300457, P. R. China;

[2] Key Laboratory of Industrial Fermentation Microbiology (Tianjin University of Science & Technology), Ministry of Education, Tianjin, 300457, P.R. China;

[3] Tianjin Key Laboratory of Industrial Microbiology, Tianjin University of Science & Technology Tianjin, 300457, P.R. China;

[4] College of Biotechnology, Tianjin University of Science & Technology, Tianjin 300457, China;

[5] College of Marine and Environmental Sciences, Tianjin University of Science & Technology, Tianjin 300457, P. R. China.

Corresponding Authors

*F. Liu, Phone: +86-22-60602717; Fax: +86-22-60602298; E-mail: fufengliu@tust.edu.cn;

*F. Lu, Phone: +86-22-60602271; Fax: +86-22-60602298; E-mail: lfp@tust.edu.cn

Table S1. The nucleotide sequence of $(NANP)_3$-TEV-$A\beta_{42}$ and primers used in amplification PCR

name	5’-3’
$(NANP)_3$-TEV-Aβ42	AATGCCAATCCGAATGCCAACCCGAACGCCAACCCGGAAAA<br>CCTGTACTTCCAGGATGCCGAGTTTCGCCATGATAGCGGCTA<br>TGAGGTGCACCACCAGAAACTGGTGTTCTTTGCCGAGGATGT<br>GGGCAGCAACAAAGGCGCCATTATTGGCCTGATGGTGGGTG<br>GCGTGGTGATTGCC
*Eco*RI-Aβ42-F	CGGAATTCAATGCCAATCCGAATGCCA
*Hin*dIII-Aβ42-R	CCAAGCTTTTAGGCAATCACCACGCC

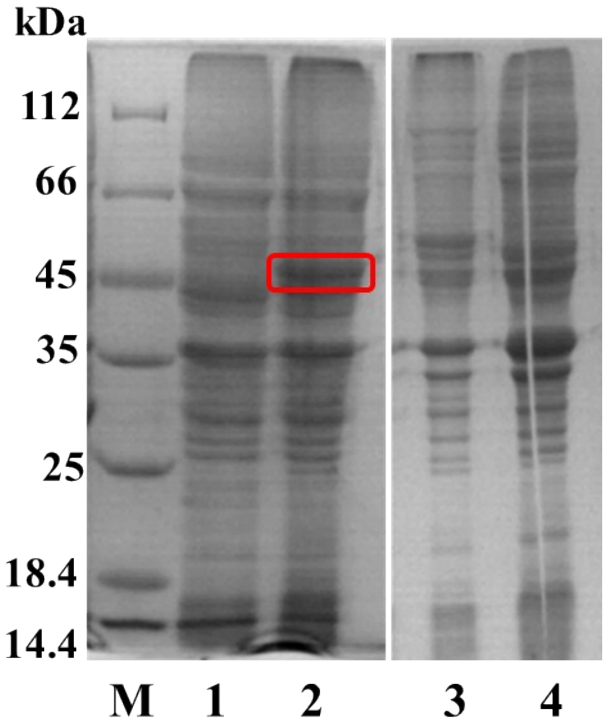

Fig S1. SDS-PAGE analysis of the soluble and insoluble fraction. BL21-MBP-Aβ42 and BL21 cells were sonicated and divided into pellet and supernatant fraction after induction by 0.5 mM IPTG. M: protein marker; 1: BL21 soluble fraction; 2: BL21-MBP-Aβ42 soluble fraction; 3: BL21 cells debris fraction; 4: BL21-MBP-Aβ42 cells debris fraction.

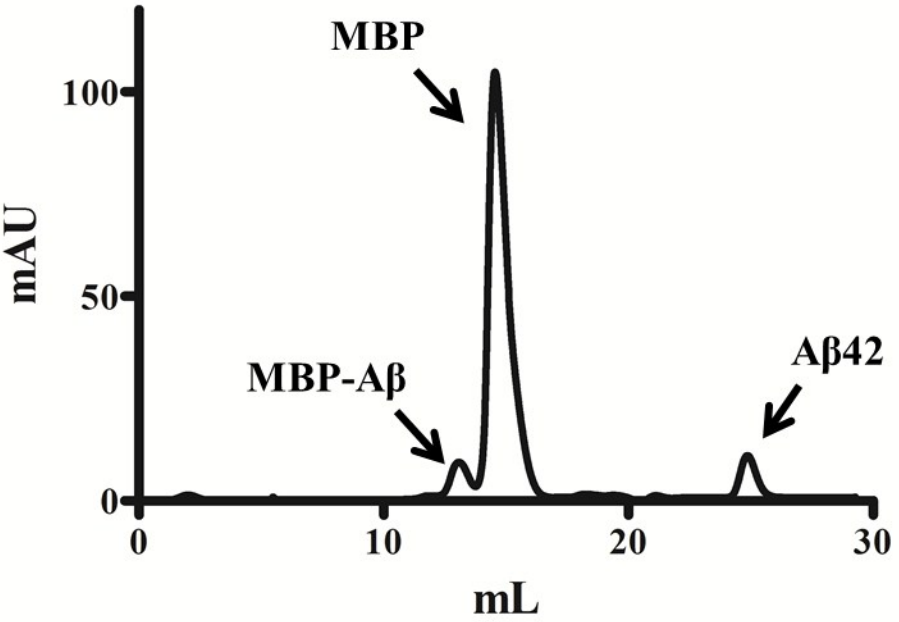

Fig S2. Chromatographic profile of the recombinant Aβ42 purified by size-exclusion chromatography on a Superdex 200 Increase column.

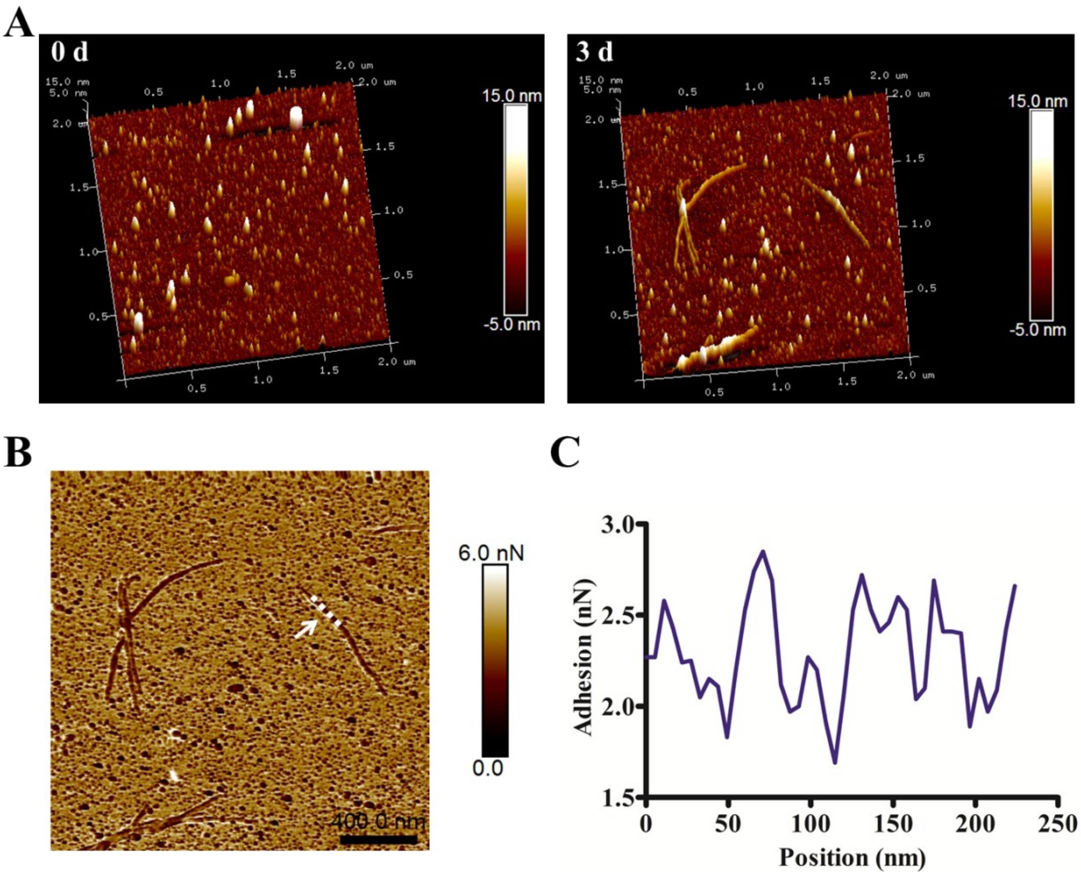

Fig S3. AFM images of recombinant Aβ42 aggregates. A: 3D images of Aβ42 aggregates after incubation for 0 and 3 days. B: AFM adhesion image of Aβ42 fibrils after 3 days' incubation. C: Adhesion profile of the dashed white line marked the position along the fibril was analyzed.
